# FAB: First UK feasibility trial of a future randomised controlled trial of Family focused treatment for Adolescents with Bipolar disorder

**DOI:** 10.1186/s40345-020-00189-y

**Published:** 2020-08-03

**Authors:** A. Sharma, M. Glod, T. Forster, R. McGovern, K. McGurk, E. Barron Millar, T. D. Meyer, D. Miklowitz, V. Ryan, L. Vale, A. Le Couteur

**Affiliations:** 1 Newcastle University, Newcastle upon Tyne, UK; 2 Cumbria, Northumberland, Tyne and Wear NHS Foundation Trust, Newcastle upon Tyne, UK; 3 Louis A. Faillace, MD, Department of Psychiatry and Behavioral Sciences, McGovern Medical School, University of Texas Health Science Center, Houston, TX USA; 4 Department of Psychiatry, Geffen School of Medicine at UCLA, Los Angeles, CA USA

**Keywords:** Bipolar Disorder, Adolescent, Randomized controlled trial

## Abstract

**Background:**

This first mixed-methods UK trial examined the feasibility and acceptability of a future definitive randomised controlled trial (RCT) to evaluate whether Family Focussed Treatment for Adolescents with Bipolar Disorder (FFT-A) UK version can improve family functioning and well-being as part of the management of Paediatric Bipolar Disorder (PBD).

**Method:**

The trial used a randomised, parallel group, non-blinded design where participants received FFT-A UK (16 sessions over 6 months) immediately or after 12 months (delayed arm). Measures of family functioning, well-being and quality of life of the young person and the main carer (most commonly a parent) were completed at baseline, 6 and 12-months in both arms. Primary outcome measures included rates of eligibility, consent and retention along with estimates of variability in the measures and assessment of the intervention delivery. Qualitative interviews allowed assessment of participants’ views about FFT-A and the trial processes.

**Results:**

Twenty-seven of 36 young persons with PBD and their families consented; of these, 14 families were randomised to the immediate and 13 to the delayed arm. Two families from the immediate arm withdrew consent and discontinued participation. Quantitative measures were completed by 22 families (88%) at 6-months and 21 families (84%) at 12-months. Qualitative interviews were conducted with 30 participants (9 young people, 15 parents and 6 other family members). Nine families attended 3 post-trial focus groups.

**Conclusion:**

It was feasible to recruit and retain to this trial. The results highlighted that trial design and measures were acceptable to participants. A benefit in family relationships was reported by participants which they attributed to the intervention in qualitative interviews. Families recommended that future modifications include definitive trial(s) recruiting participants in the age range 15–25 years as it felt this was the age range with maximum need.

*Trial registration* ISRCTN, ISRCTN59769322. Registered 20 January 2014, http://www.isrctn.com/ISRCTN59769322

## Background

Paediatric Bipolar Disorder (PBD: defined as diagnosis prior to 18 years of age) is often associated with more severe disease course, poorer functioning and impaired quality of life compared to the later onset of the disorder (Perlis et al. 2004). PBD is infrequently diagnosed in the UK (Chan et al. 2011; Stringaris et al. 2010; Sharma et al. 2016) although UK guidelines state that the peak age of onset is 15 to 19 years (NICE 2014).

Bipolar disorder impacts not only on the person affected but also their family. Our group (Barron et al. 2014) has reported significantly higher scores for intrafamilial conflict in families with Bipolar Disorder in NE England compared with healthy control families using the Family Environment Scale (Moos and Moos 1994). Belardinelli et al. (2008) have similarly reported significantly lower levels of family cohesion, expressiveness, active-recreational orientation, intellectual-cultural orientation and higher levels of conflict when comparing family environment in families with PBD to families who had no history of PBD. Further, in a US study of families with PBD, more minor conflicts occurred between family members than in either control group or families with a child with depression (Robertson et al. 2001). There is also evidence showing that parental attitudes, and in particular high levels of criticism or emotional over-involvement regarding the child—is a prospective risk factor for mood recurrences (Miklowitz et al. 2009). Furthermore, stress and the burden of caring for an ill relative are likely to increase negativity in the family environment (Casarez et al. 2019), and could in turn become a risk factor for subsequent episodes of bipolar disorder (Miklowitz and Chang 2008). Studies assessing family-based interventions which could reduce burden on the whole family as part of the long-term management of PBD are critically needed.

Psychotherapeutic studies targeting families with PBD in USA include child and family-focused cognitive-behavioural therapy (CFF-CBT) (Pavuluri et al. 2004; West et al. 2014) and Family-Focused Treatment for Adolescents (FFT-A) (Miklowitz et al. 2003). FFT-A was reported to be associated with an improvement in mood symptoms over 2 years (Miklowitz et al. 2008): PBD who received FFT-A along with medication had shorter depressive episodes and milder depressive symptoms compared to enhanced care which consisted of 3 family sessions focused on relapse prevention along with medication.

Given the low rates of diagnosis of PBD in the UK compared to USA, the cultural differences between USA and UK health care systems which prevent the direct extrapolation of published clinical effectiveness findings from USA, and in the absence of UK cost effectiveness data, the authors recognised the importance of undertaking a UK pilot feasibility and acceptability trial prior to embarking on a definitive randomised controlled trial (RCT) to evaluate the efficacy of Family Focussed Treatment for Adolescents with Bipolar Disorder (FFT-A) UK version in the UK NHS context.

### Aims of the trial

The aim of this trial was to employ a randomised, parallel group, non-blinded design allowing participants to either receive FFT-A UK (16 sessions over 6 months) immediately or after 12 months (delayed arm) to examine the feasibility and acceptability of a future definitive RCT. The specific objectives of this feasibility trial (Neely et al. 2015) were to investigate:

1. Whether it is feasible to deliver the FFT-A UK to young people with PBD (11–17 years) and their families?
2. What are the likely eligibility, consent and retention rates as well as the acceptability of being randomised to a delayed treatment arm?
3. What are young person and their family’s views about receiving the FFT-A intervention, taking part in an RCT, and completing the trial assessments and outcome measures.
4. What is the variability in the validated parent report and self-report quantitative questionnaires?

## Materials and methods

### Trial design

The feasibility and acceptability trial used a mixed quantitative and qualitative methods design with two components:

1. A single-centre, open randomised controlled trial of FFT-A UK in the management of PBD compared to treatment as usual. The primary feasibility outcomes included rates of recruitment, randomisation, retention and data completion and the variability in scores on the proposed validated questionnaires.
2. Qualitative interviews of families eligible for the intervention to investigate the acceptability of both the FFT-A UK intervention and the procedures and measures proposed for use in a definitive RCT.

### Ethical and research governance approval

The trial received a favourable ethical opinion by Sunderland NHS Research Ethics Committee (13/NE/0117) and was registered with Current Controlled Trials ISRCTN59769322. Research sponsorship was provided by Northumberland Tyne and Wear NHS Foundation Trust.

### Participants

#### Inclusion criteria

Families were eligible for recruitment into the trial if a young person in the family was between 11 and 17 years old with a confirmed diagnosis of PBD (in remission) as assessed by Washington University at St. Louis Kiddie Schedule for Affective Disorders and Schizophrenia (WASH-U-KSADS) (Geller et al. 1996). Young people and their family members needed to be prepared to be randomised, be fluent in the English language and have intellectual ability in the average range (ascertained by the referring consultant in child and adolescent psychiatry on a clinical basis), in order to complete the measures and take part in the intervention.

#### Eligibility assessment

Washington University in St. Louis Kiddie Schedule for Affective Disorders and Schizophrenia (WASH-U-KSADS) (Geller et al. 1996) is a semi-structured, clinician administered assessment of psychopathology in children and adolescents under the age of 18 years. The subject and informant version of this reliable and valid interview schedule was completed with the young person and his/her parent/carer respectively by AS who has received training on this measure at Washington University, St Louis, USA.

#### Baseline characterisation

Children’s Global Assessment Scale (CGAS) (Shaffer et al. 1983) is a clinical assessment tool that rates the general functioning of youths under the age of 18. The child or young person is given a score between 1 and 100 that relates to one of ten categories ranging from ‘extremely impaired’ (1–10) to ‘doing very well’ (91–100).

#### Demographics

Case Report Form (CRF) is a bespoke measure designed to collect demographic and medical information about the participants including data on additional diagnoses, medications taken, previous manic and depressive episodes and previous family medical history.

#### Quantitative measures

The Family Assessment Device (FAD) (Epstein 1983) is a self-report caregiver/family member 60 item questionnaire that evaluates dynamic characteristics of families and is typically completed by family members aged 12 years or older. Each item is rated on a four-point Likert scale, ranging from ‘strongly agree’ to ‘strongly disagree’. The FAD provides assessment of six domains (problem solving, communication, roles, affective responsiveness, affective involvement, and behaviour control) together with a general functioning domain that can be used as a global measure of family functioning (Byles et al. 1988). The total scale scores range from 1 to 4, with higher scores indicating unhealthy functioning. The questionnaire is reported to have good psychometric properties (Miller et al. 2000).

Conflict Behaviour Questionnaire (CBQ) (Prinz et al. 1979) is a self-report measure available in two versions (adolescent and parent) assessing problems with communication style and interpersonal behaviours within adolescent-parent dyads during the past 2 weeks. The original adolescent version has been used with both young people aged between 11 and 15 years and with older adolescents aged 12 to 17 years old (e.g. Prinz et al. 1979). The two short versions have 44 items, answerable in a true/false format and yield four scores: adolescent’s appraisal of parent, adolescent’s appraisal of dyad, parental appraisal of adolescent and parental appraisal of dyad. Each scale provides a single score, with higher scores indicating less troubled relationships.

Warwick Edinburgh Mental Wellbeing Scale (WEMWBS) (Tennant et al. 2007) is a self-report 14 items questionnaire assessing both emotional and functional aspects of mental wellbeing during the past 2 weeks in those who are 16 years old or older. The items are rated on a five-point Likert scale, ranging from ‘none of the time’ to ‘all of the time’. The questionnaire provides a single score ranging from 14 to 70, with higher scores indicating better wellbeing. The psychometric properties of the WEMWBS are robust (Lloyd and Devine 2012).

EQ-5D-L and EQ-5D-Y are standardised measures of health status designed for people aged 12 years or more and young people (aged from 5 to 18 years of age), respectively. Both versions of the measure have five dimensions: mobility, self-care, usual activities, pain/discomfort and anxiety/depression and are scored on a three-level descriptive system: no problems, some problems, and extreme problems. The measures provide also an EQ visual analogue scale (EQ VAS) that records the respondent’s self-rated health on a vertical, visual analogue scale ranging from ‘Worst imaginable health state’ to ‘Best imaginable health state’. The score can range between 0 and 100, and higher scores represent better imaginable health state.

#### Qualitative measures

In-depth qualitative interviews were conducted with participants (from both arms of the trial) after completion of 12-month follow-up. The interviews explored young peoples’ and other family members’ perspective about their participation in the trial and their experience of the FFT-A UK intervention (trial aim 3). Purposive sampling was used to achieve maximum variation across the following variables: age of young person, gender, geographic location, and family member’s relationship to the young person. A semi-structured topic guide was developed to explore the acceptability of the trial processes and experience of the intervention. Interviews were held at the family home or a nearby health centre, depending upon participant preference. As the FFT-A UK version was designed to engage families, interviews were usually conducted with more than one family member present.

#### Sample size considerations

Following published recommendations for feasibility studies (Lancaster et al. 2004), no formal sample size calculation was performed. The target sample size was 33 families with an estimated attrition rate of 10% allowing for final data analysis on 30 families thereby providing sufficient data to assess feasibility and estimate the variability in the outcome measures.

### Procedures

#### Recruitment

Participants were recruited through local community child and adolescent mental health services in North East England; the National second opinion Adolescent Bipolar Service (based in Newcastle, UK); and through an advert placed in the ‘Pendulum’, a UK national bipolar support organisation newsletter (Bipolar UK) for young people living in North East England which signposted young people to contact their local community child and adolescent mental health service. All consecutive eligible participants attending these clinical services were approached by their consultants in child and adolescent psychiatry to take part in the trial.

#### Consent

An adult family member (usually a parent) was required to give written informed consent for their own participation and for a young person aged 11–15 years in the trial. Young people aged 11–15 years gave their written informed assent whilst those aged 16–17 years gave written informed consent to participate in the trial. Upon completion of the FFT-A UK, either in the immediate or delayed arm, subsequent written informed consent and assent was taken for the qualitative interviews. Those consenting were contacted by a member of the research team to discuss a suitable date, time and venue for the qualitative interviews.

#### Randomisation and blinding

Participants were randomised to ‘immediate treatment arm’ or ‘delayed treatment arm’ in a 1:1 ratio, using random permuted blocks of sizes 2 and 4. The randomisation allocation schedule was generated by a statistician with no other involvement in the trial. Randomisation was performed by a trained member of the research team, using a secure password-protected Web-based system administered by Newcastle Clinical Trials Unit. The trial coordinator and trial statistician remained blind to allocation group. However, blinding was not feasible for the trial participants or Chief Investigator.

#### Data collection

Participants (young people and parents) completed validated questionnaires at baseline, 6- and 12-months post baseline. The completion time of the questionnaires at each timepoint was approximately 30 min. Young people and parents completed the measures which were taken to them by Clinical Studies Officers from the Clinical Research Network for North East and North Cumbria. This was to minimise contact with trial coordinator and trial statistician to prevent unblinding. Young people were given a gift voucher (to the value of £10) on completion of each batch of questionnaires.

### Intervention

#### Immediate treatment

Once randomised to immediate treatment, young people and family members either living with or involved (as defined by the young person) in the care of the young person, were invited to attend the FFT-A-UK intervention in addition to Treatment As Usual (TAU).

The FFT-A-UK consisted of 16 1-h sessions over 25 weeks at a clinical venue of the families’ choice:

- seven weekly ***psycho*-*education*** sessions focusing on the aetiology, treatment, self-management, relapse prevention plan and identifying early prodromal signs of bipolar disorder;
- four fortnightly ***communication enhancement*** sessions concentrating on teaching young people and their family members communication skills, such as offering positive feedback, active listening, making positive requests for change in others’ behaviour and giving negative feedback;
- four fortnightly ***problem*-*solving*** skill training sessions aiming to encourage an open dialogue between family members about difficult topics and help all involved to develop strategies for solving these problems;
- one final overview session completing the intervention.

#### FFT-A modification to FFT-A UK version

With DM’s (the author of the Family Focused Treatment-Adolescent (FFT-A)) agreement the FFT-A was adapted for the UK context by AS. Modifications included reducing the total number of sessions [from 21 delivered over 9 months to 16 delivered over 6 months. Some cultural modifications such as word changes were also made. The revised FFT-A UK version targets the same FFT-A key domains of psychoeducation, communication enhancement and problem-solving.

#### Therapy training

All therapists attended a 1-day training workshop on the FFT-A UK delivered by DM and AS. Throughout the feasibility trial the therapists received bi-weekly supervision from AS, and bi-monthly Skype supervision and written feedback from DM.

#### Fidelity of intervention

All therapy sessions were video-recorded and 25% were viewed by DM for purposes of fidelity monitoring, using the 11-item Therapist Competence and Adherence Scale (Weisman et al. 1998). The item scores range from 1 (very poor) to 7 (excellent).

### Data analysis

#### Quantitative outcome measures

Analyses followed a pre-specified Statistical Analysis Plan. In accordance with recommendations for the analysis of feasibility studies (Lancaster et al. 2004) analyses were descriptive only and statistical comparisons between randomised groups were not undertaken.

Eligibility, consent, and retention rates were calculated as defined. The validated questionnaires were scored according to the scoring algorithms provided (Epstein et al. 1983; NHS 2015). The scale means for the WEMWBS and subscale means for the FAD were used to impute missing items (Davidson and Mellor 2001; Beierlein et al. 2017; Bartram et al. 2011). At baseline and by randomised group the distributions of all numerical demographic and clinical variables were examined graphically and summarised by measures of location and spread. Baseline categorical variables were tabulated, and percentages reported. The questionnaire data were summarised by means and standard deviations at baseline, 6 months and 12 months for all participants with evaluable data at these time points; the purpose being to inform the design of any future definitive trials. Given the size of the trial and its feasibility objectives, no interpretation has been made of any apparent changes in the questionnaire data over time.

#### Qualitative interviews

All interviews were audio-recorded and transcribed verbatim. Analysis was conducted using anonymised transcripts. The semi-structured nature of the interviews and a priori themes meant that a largely deductive approach was taken to data coding, though the process remained flexible enough to encompass emerging issues. Codes were applied relevant to experience of trial participation and the intervention. The lead qualitative researcher (RM) conducted all interviews and coding, and initial analysis was refined following discussions with the research team (AS, MG, KM, SH) leading to the final interpretation. Findings are illustrated by exemplar quotes across all interviewees.

## Results

### Feasibility

**Fig. 1:**
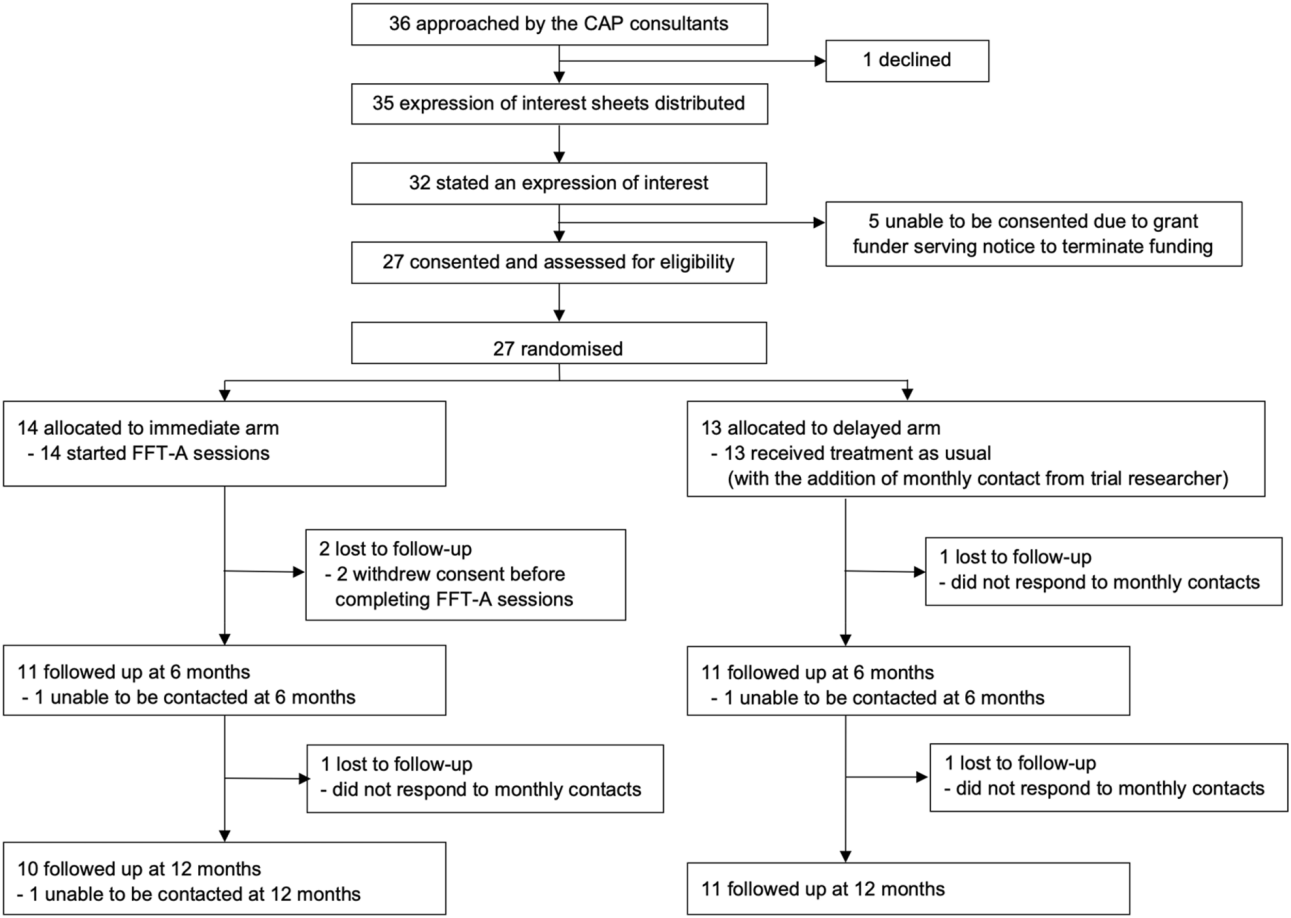
CONSORT diagram. CAP-child and adolescent psychiatry

Across the recruiting sites, 36 potentially eligible young people with PBD were approached by their Consultants in Child and Adolescent Psychiatry. All but one agreed to consider the trial. Of the 36 potentially eligible families, 32 (89%) expressed interest in participating in the trial and 27 (75%) consented to take part. An unavoidable research funding constraint (17 months into the trial) resulted in a delay of 5 months with recruitment. This delay meant that 5 young people (3 male, 2 female) and their families—although having expressed an interest in participating in the trial –no longer met the inclusion criteria when the trial resumed because the young people were over the age of 18 years. Fourteen families were randomised to the immediate treatment arm of which 2 families withdrew consent after receiving between 4 and 5 sessions of the intervention. Retention rate, defined as number of participants completing their allocated treatment, was 24/27 families (89%). Twelve families (range of 2–5 family members) completed the treatment in the immediate arm, attending between 12 and 16 sessions (median = 15.5) over between 3 months 29 days to 10 months 16 days (median 6 months 5 days). At 6 months the measures were completed by 11 families in both the immediate and the delayed arms, and at 12 months by 10 families in the immediate arm and 11 families in the delayed arm (Fig. 1).

### Baseline demographic and clinical characteristics

At baseline the two groups were balanced in terms of demographic and clinical characteristics (see Tables 1 and 2). Questionnaires were completed by 22 (88%) of the families at the 6 months and 21 (84%) at 12 months.

**Table 1:** Participant & family demographic and clinical characteristics at recruitment

	Immediate arm, n = 14	Delayed arm, n = 13	Total, n = 27
Age in years: mean (sd)	16.2 (1.3)	16.1 (1.5)	16.1 (1.4)
Index of Multiple Deprivation: mean (sd)	23.4 (14.8)	23.2 (11.1)	23.3 (12.9)
CGAS rating: mean (sd)			
Current (last 2 weeks)	84 (7)	81.4 (5.2)	82.7 (6.3)
Most severe past episode	25.1 (5)	22.4 (8.1)	23.8 (6.8)
Highest in prior year	81 (7)	81.5 (5)	81.2 (7)
Gender: n (%)			
Female	13 (92.9)	10 (76.9)	23 (85.2)
Ethnicity: n (%)			
White British	13 (92.9)	12 (92.3)	25 (92.6)
White other	0 (0)	1 (7.7)	1 (3.7)
Mixed	1 (7.1)	0 (0)	1 (3.7)
Type of bipolar disorder: n (%)			
Bipolar I	8 (57.1)	7 (53.8)	15 (55.6)
Bipolar II	6 (42.9)	5 (38.5)	11 (40.7)
Bipolar NOS	0 (0)	1 (7.7)	1 (3.7)
Comorbid disorders			
GAD	0 (0)	2 (15.4)	2 (7.4)
ASD	2 (14.3)	2 (15.4)	4 (14.8)
ADHD	1 (7.1)	0 (0)	1 (3.7)
ASD + ADHD	1 (7.1)	0 (0)	1 (3.7)
Substance abuse or dependence disorder (past 3 months)	0 (0)	0 (0)	0 (0)
Mood episodes			
Age of diagnosis of BD in years mean (sd)	12.8 (1.6)	12.9 (1.3)	12.8 (1.4)
Hypomania			
Number of participants n (%)	8 (57.1)	8 (61.5)	16 (59.3)
Number of episodes mean (sd)	3 (1.1)	2.9 (2)	3 (1)
Lifetime duration in weeks mean (sd)	7.8 (4.4)	8.6 (4.5)	8.1 (4.3)
Admissions to hospital	0 (0)	0 (0)	0 (0)
Mania			
Number of participants n (%)	8 (57.1)	7 (53.8)	15 (55.6)
Number of episodes mean (sd)	2.9 (0.8)	2.7 (0.8)	2.8 (0.8)
Lifetime duration in weeks mean (sd)	13.4 (5.2)	14 (5.3)	13.7 (5.1)
Number of participants experiencing psychotic episodes n (%)	5 (35.7)	4 (30.1)	9 (33.3)
Admissions to hospital n (%)	4 (30.1)	3 (23.1)	7 (25.9)
Depression			
Number of participants n (%)	14 (100)	13 (100)	27 (100)
Number of episodes mean (sd)	4.2 (1.1)	4.2 (1.1)	4.2 (1.1)
Lifetime duration in weeks mean (sd)	37.2 (16.9)	35.9 (16.2)	36.6 (16.5)
Number of participants experiencing psychotic episodes n (%)	10 (71.4)	9 (69.2)	19 (70.4)
Admissions to hospital n (%)	7 (50)	6 (46.2)	13 (48.1)
Medication treatments at trial entry			
Mood stabilizer	5 (35.7)	5 (38.5)	10 (37)
Antipsychotic	8 (57.1)	10 (76.9)	18 (66.7)
Other	1 (7.1)	0 (0)	1 (3.7)
None	4 (28.6)	0 (0)	4 (14.8)
Family medical history (first degree relatives)			
Bipolar disorder	5 (35.7)	4 (30.8)	9 (33.3)
Depression	4 (28.6)	10 (76.9)	14 (51.9)
Anxiety	2 (14.3)	1 (7.7)	3 (11.1)
ADHD	2 (14.3)	3 (23.1)	5 (18.5)
ASD	2 (14.3)	2 (15.4)	4 (14.8)
Physical illness	1 (7.1)	0 (0)	1 (3.7)
Other	2 (14.3)	3 (23.1)	5 (18.5)

*CGAS* Children’s Global Assessment Scale, *NOS* not otherwise specified, *GAD* generalised anxiety disorder, *ASD* autism spectrum disorder, *ADHD* attention deficit hyperactivity disorder

**Table 2:** Quantitative outcome measure summaries at each time point and by randomised arms

Outcome measure	Baseline		6-month follow-up		12-month follow-up	
*n* mean (sd)	Immediate arm	Delayed arm	Immediate arm	Delayed arm	Immediate arm	Delayed arm
WEBWBS^a^	14 43.6 (8.6)	13 40.2 (8.9)	11 44.6 (9.4)	11 40.8 (9.3)	10 45.5 (9.2)	11 43.0 (6.7)
FAD^b^	14	13	11	10	9	10
Problem solving	2.0 (0.5)	2.3 (0.5)	1.8 (0.3)	2.1 (0.4)	1.9 (0.5)	2.2 (0.3)
Communication	2.2 (0.4)	2.4 (0.6)	2.0 (0.4)	2.3 (0.5)	2.0 (0.4)	2.2 (0.5)
Roles	2.5 (0.3)	2.5 (0.4)	2.3 (0.4)	2.6 (0.5)	2.4 (0.3)	2.5 (0.5)
Affective responsiveness	2.0 (0.6)	2.4 (0.8)	1.8 (0.5)	2.3 (0.7)	1.7 (0.5)	2.2 (0.6)
Affective involvement	2.3 (0.4)	2.3 (0.4)	2.1 (0.4)	2.3 (0.3)	2.2 (0.5)	2.3 (0.5)
Behaviour control	2.0 (0.3)	2.0 (0.6)	1.7 (0.3)	1.9 (0.5)	1.8 (0.3)	1.7 (0.5)
general functioning	2.1 (0.4)	2.2 (0.6)	1.8 (0.3)	2.4 (0.6)	1.7 (0.4)	2.2 (0.3)
CBQ-parent^c^	14	13	11	10	9	10
Appraisal of young person	13.0 (9.4)	13.2 (6.8)	7.0 (6.2)	13.8 (7.0)	5.8 (5.4)	10.5 (8.6)
Appraisal of dyad	4.6 (4.1)	4.6 (3.5)	1.5 (1.9)	5.7 (4.1)	2.6 (2.9)	6.1 (6.7)
CBQ-adolescent^c^	14	13	11	11	10	11
Appraisal of parent	10.6 (9.2)	7.0 (6.3)	5.3 (7.4)	5.6 (5.6)	5.3 (7.0)	8.0 (6.0)
Appraisal of dyad	5.2 (4.4)	3.7 (3.4)	2.5 (2.9)	4.5 (3.0)	3.0 (3.3)	3.6 (3.9)
EQ-5D-Y *n* Frequency						
Mobility	14	13	11	11	10	11
No problems	13	11	10	9	9	8
Some problems	1	2	1	2	1	3
A lot of problems	0	0	0	0	0	0
Self care	14	13	11	11	10	11
No problems	13	11	10	10	9	9
Some problems	1	2	1	1	1	2
A lot of problems	0	0	0	0	0	0
Performing usual activities	14	13	11	11	10	0
No problems	11	4	9	3	5	5
Some problems	2	9	2	5	5	4
A lot of problems	1	0	0	2	0	2
Pain or discomfort	14	13	11	11	10	11
No problems	8	7	8	7	6	6
Some problems	6	4	3	4	3	5
A lot of problems	0	2	0	0	1	0
Feeling worried, sad or unhappy	14	13	11	11	10	11
No problems	8	4	8	4	5	2
Some problems	4	7	3	3	4	8
A lot of problems	2	2	0	4	1	0
EQ-5D-3L *n* Frequency						
Mobility	14	12	11	10	9	10
No problems	14	12	11	10	8	10
Some problems	0	0	0	0	1	0
A lot of problems	0	0	0	0	0	0
Self care	14	12	11	10	9	10
No problems	14	12	11	10	9	10
Some problems	0	0	0	0	0	0
A lot of problems	0	0	0	0	0	0
Performing usual activities	14	12	11	10	9	10
No problems	13	8	10	9	9	7
Some problems	1	4	1	1	0	3
A lot of problems	0	0	0	0	0	0
Pain or discomfort	14	12	11	10	9	10
No problems	10	9	9	8	6	6
Some problems	4	3	1	2	2	4
A lot of problems	0	0	1	0	1	0
Feeling worried, sad or unhappy	14	13	11	11	10	11
No problems	5	4	9	5	7	3
Some problems	8	7	2	4	2	4
A lot of problems	1	1	0	1	0	3

^a^WEBWBS: higher score indicates better well-being

^b^FAD: higher score indicates unhealthy functioning

^c^CBQ: higher score indicates more conflict

### Fidelity monitoring of psychotherapy

The average overall adherence/competence rating was 4.18 (SD = 0.40; range 1–7) indicating a good level of adherence on the TCAS scale.

### Quantitative measures

The WEMWBS, FAD, CBQ-parent and CBQ-adolescent were examined for completion. Data quality was high with no missing items for the CBQ-parent and CBQ-adolescent and only a small number of missing items for the WEMWBS and the FAD so that an overall score could be calculated according to the scoring algorithm (Table 2).

### Health economics

The responses to the EQ-5D-Y appeared to suggest that young people experienced relatively few problems with mobility and with looking after themselves compared with the other questions. A similar pattern was found for carers. With respect to the visual analogue score, as would be expected there was considerable variation between respondent both for the young people and carers.

### Qualitative findings

Nine young people M:F: 1:8), 15 parents (Fathers: Mothers: 12:3) and 6 other family members (n = 3 siblings, n = 2 grandmothers, n = 1 partner) consented to be interviewed. Eighteen interviewees were from the immediate arm group (6 young people, 8 mothers, 1 father, 1 sibling, 2 grandmothers) and twelve were from the delayed arm (3 young people, 4 mothers, 2 fathers, 2 siblings, 1 partner). The average number of interviewees present in the interviews was two (range 1–5). The average duration of the interviews was 1 h and 5 min (range 29 min–1 h and 26 min).

### Acceptability of intervention and research processes and procedures

Participating young people and their family members spoke positively about the trial. Typically, a view was shared that research was important and necessary to identify effective interventions for young people diagnosed with PBD. Moreover, the inclusion of the family in both the research and the intervention itself was welcomed, with participants feeling that it was appropriate to focus upon the family. Young people and their families reported that they had been very happy to be involved in the trial and had understood what they were consenting to participate in. Whilst participant motivation to consent to the trial was typically to access intervention, participants understood and were accepting that they may randomly be assigned to the delayed treatment arm. As such, the trial procedures were found to be acceptable to the trial population.

*[Psychiatrist’s name] asked if we would do it and anything, absolutely anything that will give us some insight. Because there was nothing there. Nothing, absolutely nothing (Mother of 16*-*year*-*old son).*

Participants were accepting of baseline and follow-up assessment. Indeed, all trial processes were considered to be appropriate and participants reported being willing to comply.

### Experience of intervention

The majority of participants found the educational components of the FFT-A UK to be helpful, reporting that the information was useful in promoting understanding and acceptance of the young person’s diagnosis. Both young people and their family members reported feeling reassured by the information, and the knowledge that others share some of their symptoms and experiences.

*I remember they showed us a video of all these people in America who had had bipolar, and I got to a point where I was like was I doing all of that for attention…but then I watched them and my God, I realised it must really be bipolar. That’s when I think I finally accepted it (female aged 17* *years).*

The communication and problem-solving sessions were also generally well received, with a number of the participants reporting that they had experienced benefit from them. The opportunity to sit down as a family and talk about challenges they experienced with one another was valued by participants.

*I think they actually helped [us] to communicate better, because there were a lot of times when we wouldn’t say what we were actually feeling to each other (female age 17* *years).*

### Impact of the intervention

Many of the participants reported that they felt that the intervention had a positive impact upon them. For some, the intervention had helped them to understand the young person’s diagnosis, and how it affected them, enabling them to better manage their symptoms.

*I know who I am now, and how I work, and what I can do to help myself, instead of just letting things get really bad again (female age 15* *years).*

For others, the benefit they perceived from the intervention related to the quality of the family relations. Improved relationships with siblings in particular were discussed by participants. An increased understanding the young person’s diagnosis was reported to positively affect relationships. The combination of factual information and an opportunity for the family to talk openly resulted in increased understanding between family members. This was often discussed in relation to siblings, for whom the young person’s behaviour was often viewed as ‘attention seeking’ or resulting in ‘unfair lenience’ from the parents.

*The FAB study helped…[Young person’s brother] used to say, “Well, yes, she just wants more attention. She’s this. She’s that.” When he actually realised, he has a great deal of empathy. He can tell when she is playing me as opposed to illness, and he’s better at that than I am (mother of 15* *year old daughter).*

*We have sister days and everything now (sister of 17* *year old female).*

Some families reported that they had experienced improved family functioning following their involvement in the FAB intervention. Participants discussed strategies they had developed to manage interactional difficulties or strains within the family home. Improved communication was also highlighted.

*It’s more like we’re a team (father of 16*-*year*-*old daughter).*

A number of participants however reported that whilst they found the sessions informative, they were not helpful in affecting change. This tended to relate to a difficulty in putting the lessons into practice outside of the sessions.

*Communication skills, I thought it was brilliant. But every time I tried to do that, I got shot down in flames (Father of 16*-*year*-*old female).*

*Really informative but when you’ve got a kid that doesn’t communicate, again, it doesn’t work (mother of 16*-*year*-*old son).*

For a significant minority however, the sessions were highlighted as not sufficient to tackle what they considered to be substantial or complex difficulties. For some of the participants there was a sense that one must be realistic about the potential impact of a talking therapy, and that only small benefits could be expected. Others reported that the sessions were too basic to meet their needs resulting in disappointment.

*What any parent or partner of somebody with bipolar would want is a cure. There’s no cure. Therefore, anything is a little bit of a toothless tiger (mother of a 15*-*year*-*old daughter).*

*The problems with my friends couldn’t be solved. I failed some of my classes, they can never be solved. I lost my dad, they can never be solved. (female aged 17* *years).*

### Improvements to the intervention

The timing of the intervention was considered by many to be very important. After receiving a diagnosis, many young people and their families had often made significant effort to learn about PBD before attending the sessions, feeling in need of immediate and detailed information. As such, participants reported that they often received the intervention ‘too late’.

*There is not point doing the sessions once person’s already been through all the crap …but if FAB was there sooner, maybe none of that would have happened (female aged 17* *years).*

Participants were mostly very positive about the skills of the practitioners delivering the intervention. These families spoke highly of the knowledge and expertise of the therapists, considering their attributes to be fundamental to their positive experience of the intervention. For a minority, however, the perceived lack of therapist skill was a major issue.

*But the problem with the communication around the family and the problem solving is that [practitioner’s name] is a brilliant case worker for [young person’s name] but she was out of her depth managing the family dynamic. So we had one session like that, which was fairly disastrous (father of a 12* *year old son).*

### Post-trial focus groups

As part of the trial, all families participating in the research were invited to a series of 3 workshops focussing on sharing of research findings with participants and the planning of a subsequent definitive RCT. The participants from 9 families who attended (on average n = 19 (young people and parents across the 3 workshops) felt that the research findings resonated with them. In planning a following trial participants made a number of recommendations. They felt that ‘when’ this type of intervention is offered to families is crucial; if it is too early post diagnosis, the family is likely to still be in the process of acceptance and if too late the family might have accessed many aspects of the intervention elsewhere reducing the potential benefits of the intervention. The families agreed that the ideal time would be between 3 months and 18 months post diagnosis. They also emphasised the need for the therapist not to be the same person offering routine care for the young person as this had occurred for some participants and was not well received. Both young people and their family members recommended that the FFT-A UK should be offered should be 15–25 years rather than just under 18 years. They highlighted that this was the time of greatest need in light of multiple transitions such as moving to adult mental health services, starting college/university and/or living independently.

## Discussion

Despite the previously reported rates of infrequent diagnosis of PBD in the UK (Stringaris et al. 2010; Sharma et al. 2016) this trial has demonstrated that an efficacy trial is likely to be feasible and able to recruit successfully to target from UK community based child and adolescent mental health services. Further the intervention and the research protocol were found to be acceptable to the families who consented to take part in this pilot trial. The trial successfully recruited 81% of the target sample although recruitment took longer than originally anticipated (i.e. 18 months instead of 12 months). In terms of retention only two families (7.4%) dropped out shortly after randomisation which is a lower attrition rate than reported in previous US studies (17.2% 34, 15.2% 35).

It is relevant to note that adults with BD often indicate that their primary concerns are about the adverse impact that the illness has had on their psychosocial functioning such as family and social relationships, education or financial independence. As a consequence, they rated their social support networks as a more important factor determining their quality of life than their mental health (Michalak et al. 2006). These findings suggest that *functional as well as symptomatic* recovery (Colom and Vieta 2004) should be targeted in treatments for BD. Consideration of these wider impacts have been shown to be especially important for young people with PBD and their families (Gomes et al. 2016; Freeman et al. 2009; Lofthouse and Fristad 2004). In our trial questionnaire completion rates were also encouraging and similar to previously published studies, where 82.8% of the participants completed 1-year follow-up (Miklowitz et al. 2008) and 84.4% of the participants had at least one follow-up interview (Miklowitz et al. 2014). The high rates of questionnaires completion not only indicate families’ commitment to and their acceptance of the research process but also an appropriate choice of quantitative measures which were selected after extensive Patient and Public Involvement and Engagement (PPIE). Preparatory focus groups with families attending the National Specialist Adolescent Mood disorders Service (SAMS) based in Northeast England, as part of the Public and Patient Involvement to help develop the objectives for this project, identified that young people with PBD and their families wanted help with understanding what PBD means for them, how they might relate to each other in a better way and how they could seek help when the clinical situation deteriorated. They highlighted that their top priority was the need to assess the impact of any new non-pharmacological intervention on family functioning rather than just focussing on symptomatic recovery of the young person with PBD.

Although the participants in this trial were similar in age (mean = 16.1, SD = 1.4) to those recruited for the Miklowitz et al. (2008) FFT-A randomised trial (mean = 14.5, SD = 1.6; n = 58), the ratio of girls who took part in the UK FAB trial was higher (85.2% compared to 56.9%). This high percentage of female participants was surprising since the male to female ratio in bipolar disorder is equal (Blehar et al. 1998). The higher percentage of girls referred to the trial team was out with the research team’s control. Interestingly the ratio of young men and women who were approached and eventually consented to take part in the trial were similar.

These research findings suggested that participants were very supportive of the research, believing it to be important and necessary to progress effective interventions for young people with PBD and their families. Participants also found trial processes to be acceptable. The intervention was largely well received, with many young people and their families reporting a benefit which they attributed to the FAB intervention. There were mixed views however on the usefulness of the various components of the intervention, as well as the duration. These findings highlight the importance of individualised therapy for the treatment of YP with PBD as recommended in NICE guidelines (NICE 2014). Although a personalised approach is recommended in the current FFT-A UK training manual and sessions, however, some further modification such as the inclusion of core sessions and additional, optional sessions were recommended by the families. This might enable FAB therapists and the family to work together to identify the content and depth most relevant to the family, whilst also avoiding excessive burden of time.

### Next steps

In keeping with the findings of the post-trial focus groups, a definitive RCT assessing the clinical and cost- effectiveness of FFT-EOY is being planned. Although such a trial will benefit from the experience of completing this feasibility and acceptability study, it will target a somewhat older age range (15–25-year olds: older adolescents and young adults) who have been recently diagnosed with BD (between 3 and 18 months earlier). To date, Family Focussed Treatment (FFT) and FFT-A have been used across 8 randomized controlled trials with adults and adolescents with BD. Combined with medication, FFT has been associated with more rapid recovery from mood episodes, a lower rate of recurrences, and lower levels of symptom severity compared to briefer forms of psychoeducation and medications over 1–2 years (Miklowitz and Chung 2016). Miklowitz et al. have developed an adapted version of the intervention targeting 13–25 years of age (FFT-EOY (2008)) which our group has already adapted to the UK cultural context. Therefore, we anticipate the future definitive trial having an impact on both young adults and older adolescents. Furthermore, targeting this age group will increase the number of individuals available for the trial and assist in achieving adequate power to observe definitive effects. The future definitive trial will contribute to a UK evidence base evaluating not only the clinical effectiveness but also the cost-effectiveness of a manualised psychological intervention for BD in the context of the UK National Health Service (NHS).

### Strengths and limitations

This evaluation of an RCT assessing the feasibility and acceptability of FFT-A UK version in the management of PBD and further focus groups with participants suggest that recruitment to a definitive trial assessing effectiveness and cost-effectiveness of a similar intervention in the age range 15–25 years would be possible. In our trial, although the initial recruitment was slower than anticipated (a common finding in intervention trials], once the trial became more widely known, the resulting momentum allowed recruitment to 81.8% of target sample and retention of 92.6% of consented sample.

To our knowledge this is the first UK trial of FFT-A UK for young people with PBD which has included an embedded qualitative trial. Our research included young people, their parents as well as other key family members, therefore gathering a range of views. We purposively sampled male family members, often underrepresented in research involving parents which typically gathers the views of mothers. Despite our efforts, most of our sample was female, a trial limitation. Further, we were only able to interview young people and participants who consented to join the research trial investigating the feasibility and acceptability of a psychoeducational intervention. The trial did not include measures of mood severity as it was felt that this would not be relevant to the trial aims but a definitive trial will include these as outcome measures.

## Conclusions

It was feasible to recruit participants to this feasibility and acceptability trial. Furthermore, retention rates were also satisfactory in this trial. The results highlighted that trial design and measures were acceptable to participants. A benefit in family relationships was reported by participants which they attributed to the intervention in qualitative interviews. Families made recommendations for future modifications. Focus groups recommended that the future definitive trial recruit participants in the age range 15–25 years (between 3- and 18-months post diagnosis) as it felt this was the age range with maximum need. Given the positive demonstrated benefits for both adults and adolescents with age-appropriate versions of FFT, we think this age range would be a suitable approach to definitively study clinical- and cost-effectiveness of the FFT-EOY.

## Data Availability

The datasets used and/or analysed during the current trial are available from the corresponding author on reasonable request.
